# The mitochondria-independent cytotoxic effect of nelfinavir on leukemia cells can be enhanced by sorafenib-mediated mcl-1 downregulation and mitochondrial membrane destabilization

**DOI:** 10.1186/1476-4598-9-19

**Published:** 2010-01-27

**Authors:** Ansgar Brüning, Martina Rahmeh, Andrea Gingelmaier, Klaus Friese

**Affiliations:** 1 Ludwig-Maximilians University Munich, Department of Obstetrics/Gynaecology, Molecular Biology Laboratory, Munich, Germany

## Abstract

**Background:**

Nelfinavir is an HIV protease inhibitor that has been used for a long period of time to treat HIV-infected individuals. It has recently emerged that nelfinavir could represent a prospective new anti-cancer drug, prompting us to test the effect of nelfinavir on leukemia cells.

**Methods:**

By combining *in vitro *and *ex vivo *studies, the effect of nelfinavir on leukemia cells and non-malignant, bone marrow-derived tissue cells was analyzed.

**Results:**

At a concentration of 9 μg/ml, nelfinavir induced death of 90% of HL60, IM9, and Jurkat cells. At the same concentration and treatment conditions, less than 10% of aspirated human bone marrow cells showed nelfinavir-induced cell damage. Nelfinavir-induced death of leukemia cells was accompanied by activation of caspases 3, 7, and 8. Despite caspase activation, the upregulation of the anti-apoptotic bcl-2 family member protein mcl-1 that resulted from nelfinavir treatment stabilized the mitochondrial membrane potential, resulting in primarily mitochondria-independent cell death. Pharmacological downregulation of mcl-1 expression by treatment with sorafenib (2 μg/ml) significantly enhanced nelfinavir-induced apoptosis even at lower nelfinavir concentrations (5 μg/ml), but did not have additional detrimental effects on non-malignant bone marrow cells.

**Conclusions:**

The ability of nelfinavir to induce apoptosis in leukemia cells as a single agent in a mitochondria-independent manner might suggest it could be used as a second or third line of treatment for leukemia patients for whom standard mitochondria-directed treatment strategies have failed. Combination treatment with nelfinavir and sorafenib might further enhance the efficacy of nelfinavir even on chemo-resistant leukemia cells.

## Background

Blood cancer cells are highly sensitive to cytostatic drugs but, depending on the cancer type, often become resistant after initial therapy, necessitating second and even third line treatment therapies. Thus, there is a need for additional new anti-cancer drugs that induce specific cell death pathways in leukemia cells. It has recently been shown that the HIV protease inhibitor nelfinavir (Viracept^®^) can induce cell death in a variety of human cancer types [1,2], and clinical studies with nelfinavir are currently proposed or underway [3-6]. Nelfinavir appears to induce cell death in human cancer cells by rather pleiotropic mechanisms, including apoptosis, necrosis, and autophagy [1,2]. Swelling of the endoplasmic reticulum by an accumulation of misfolded proteins (ER stress response) appears to be a central mechanism in nelfinavir induced death in several cancer types, including lung cancer [1], glioma [2], and ovarian cancer cells [7,8], and precedes the activation of apoptosis.

Apoptosis can be induced by several pathways, including an extrinsic pathway mediated by cell membrane-bound death receptors and an intrinsic pathway mediated by activation of pro-apoptotic intracellular mechanisms [9]. Mitochondria play a central role in the induction and control of apoptosis because they harbour several apoptosis-inducing proteins within their membranes that can be released into the cytosol (cytochrome c, smac/DIABLO) to induce caspase-dependent cell death [9,10]. Release of these mitochondrial factors occurs via outer mitochondrial membrane pore formation by pro-apoptotic bcl-2 family members, such as bax, bak and t-bid. The activities of these pro-apoptotic molecules are counterbalanced by the anti-apoptotic mitochondrial membrane proteins bcl-2, bcl-XL, and mcl-1 [9-12]. Although there are several different theories regarding how the pro- and anti-apoptotic bcl-2 family members interact [11,12], it has repeatedly been shown and is generally believed that increased expression of pro-apoptotic bcl-2 family members promotes cell death, whereas increased expression of anti-apoptotic bcl-2 family members facilitates cell survival. The most prominent anti-apoptotic bcl-2 family members, including bcl-2 (B-cell CLL/lymphoma 2), bcl-XL (BCL2L1) and mcl-1 (myeloid cell leukemia 1; BCL2L3), were originally identified and found to be over-expressed in leukemia cells [13,14]. Mcl-1 is a rather unique member of the bcl-2 family in that it has a relatively large molecular weight of 40/42 kDa, compared to the molecular weight of ca. 26 kDa common to most other bcl-2 family members. Mcl-1 is a target of several pro-apoptotic proteins and has been shown to undergo caspase-mediated degradation during apoptosis [15]. Further, a shorter splice form of mcl-1 (mcl-1s; 36 kDa) has been described and has been shown to exert a pro-apoptotic function [16]. Thus, expression and modification of mcl-1 appears to be crucial for regulation of cell survival and cell death in leukemia cells [17,18]. In the present study, we show that despite its ability to induce apoptosis, nelfinavir enhances expression of the mitochondria-protective mcl-1 protein in leukemia cells, resulting in a primarily mitochondria-independent caspase activation and cell death.

## Methods

### Cells and cell culture

The human leukemia cell lines Jurkat (acute T-cell leukemia, ATCC TIB-152), HL-60 (acute promyelocytic leukemia, ATCC CCL-240) and IM-9 (EBV-transformed B-lymphoblastoid, ATCC. CCL 159) were cultured in RPMI-1640 medium supplemented with 10% fetal calf serum and antibiotics at 37°C in a humidified atmosphere with 5% CO_2_. All cell culture reagents were from PAA, Pasching, Austria. Stromal bone marrow cells, enriched by Ficoll gradient centrifugation as described [19], were kindly provided by the Tumour Immunology Department of the University Hospital, Munich. Bone marrow fibroblasts were generated by allowing bone marrow cells to adhere to plastic cell culture flasks. Cells were grown for 4 weeks, and non-adherent cells were regularly displaced by replacing the cell culture medium. Cells exhibited a typical fibroblast-like morphology, and fibroblasts appeared to be the only cell type from bone marrow cells that showed significant proliferation under the cell culture conditions used.

### Drugs and drug treatment

Nelfinavir mesylate (Viracept^®^; MW 663,90) was generously provided by Pfizer, Groton, CT, USA. Nelfinavir was dissolved in DMSO and stored at -20°C as a 50 mg/ml stock solution. The primary concentration used in this study was 8 μg/ml nelfinavir mesylate, corresponding to a molar concentration of 12 μM. Sorafenib (Axxora, Lörrach, Germany) was stored as a 25 mg/ml stock solution in DMSO. In control experiments, cells received an amount of DMSO equal to that used in the treated cells. Staurosporine (Sigma, Munich, Germany) was stored as a 500 μM stock solution in DMSO.

### Chemosensitivity assay (ATP-TCA assay)

To test the viability of the cancer cells, 5000 cells in a total volume of 200 μl were plated in flat-bottomed 96-well plates (Nunc, Wiesbaden, Germany) and incubated with nelfinavir for 48 h at 37°C. For cell extraction, 50 μl tumour cell extraction buffer (DCS Innovative Diagnostik-Systems, Hamburg, Germany) was added to each well, mixed thoroughly, and incubated for 20 minutes at room temperature. Using a MicroLumat LB 96P bioluminometer (EG&G Berthold, Bad Wildbad, Germany), Luciferin-Luciferase agent (DCS Innovative Diagnostik-Systems, Hamburg, Germany) was added automatically to each sample and samples were analyzed for bioluminescence.

### Annexin binding assay

FITC-labelled annexin V (Biocat, Heidelberg, Germany) was added to viable cells as recommended by the supplier in combination with propidium iodide, and cells were analyzed with a FACScan using an FL-1 setting (propidium iodide) at 575 nm and an FL-2 setting (FITC) at 530 nm. FACScan analysis was performed using a Becton Dickinson FACScan analyzer (Becton Dickinson, Heidelberg, Germany).

### Cell cycle analysis

For cell cycle analysis, leukemia cells were washed with phosphate-buffered saline (PBS), fixed with 70% methanol, incubated with RNase (Sigma, Munich, Germany), and stained with propidium iodide prior to FACScan analysis (575 nm filter).

### Mitochondrial membrane potential analysis

To analyze the mitochondrial membrane potential, the MitoCapture Mitochondrial Apoptosis Detection Kit (Axxora, Lörrach, Germany) was used according to the manufacturer's instructions. For FACScan analysis, an FL-1 setting (red fluorescence) at 575 nm and an FL-2 setting (green fluorescence) at 530 nm were used. Similar filters were used for fluorescence microscopy.

### Western blot analysis

Western blot analysis was performed as recently described [8]. Cell extracts were prepared with RIPA-buffer (50 mM Tris, pH 8.0, 150 mM NaCl, 1% NP40, 0.5% doxycholine, 0.1% SDS), and 20 μg of protein (BioRad Bradford Assay, BioRad, München, Germany) was subjected to SDS-polyacrylamide gel electrophoresis. Proteins were transferred to PVDF membranes in a BioRad Mini Protean II Cell (BioRad, Munich, Germany) at 1 mA/cm^2 ^membrane in 10% methanol, 192 mM glycine, and 25 mM Tris, pH 8.2. Membranes were blocked with 4% non-fat milk powder in PBS-0.05% Tween for 4 h. Primary antibodies were applied in blocking buffer and incubated at room temperature overnight. Antibodies against caspases and ER stress-related proteins were included in antibody sampler kits purchased from Cell Signalling, NEB, Frankfurt, Germany. Polyclonal antibodies against PARP, bak, bid, bcl-XL, LC3, and COX IV were purchased separately from Cell Signalling (NEB, Frankfurt, Germany). Antibodies against ATF3, β-actin, BiP, mcl-1, and p53 (DO1) were from SantaCruz Biotech (Heidelberg, Germany). Monoclonal cell cycle regulatory antibodies were included in a cell cycle antibody sampler kit from BD Biosciences, Heidelberg, Germany.

### RT-PCR analysis

RNA was extracted from cells using the Nucleospin RNA II kit (Macherey-Nagel, Düren, Germany). Reverse transcription was performed with M-MLV reverse transcriptase (Promega, Mannheim, Germany), as recommended by the supplier. PCR was carried out in an Eppendorf Mastercycler with GoTaq (Promega, Mannheim, Germany). Primer pairs (5'-3' gccggctgtc ctgccgctgc/tta cagtaaggctatcttattag) were used to amplify a 402 bp C-terminal fragment of mcl-1 (MCL1S) and a 640 bp fragment (MCL1L). The difference between MCL1S and MCL1L is generated by alternative splicing within this region [16]. PCR cycling was performed after a 5 min initiation at 94°C with 26-28 cycles of 1 min at 94°C, 1 min at 57°C, and 2 min at 72°C, followed by a 5 min extension at 72°C.

### Mitochondria isolation

Cells were collected by centrifugation at 750 *g *for 5 min, washed once with PBS (pH 7.4), and resuspended in five volumes of buffer A (250 mM sucrose, 20 mM HEPES, 10 mM KCl, 1.5 mM MgCl_2_, 1 mM EDTA, 1 mM EGTA, 1 mM dithiothreitol, 0.1 mM pefabloc, pH 7.5) as described [20]. The cells were homogenized in a 2-ml glass Dounce homogenizer (VWR, Darmstadt, Germany) using the loose fit pestle for 4 strokes and the tight fit pestle for an additional 10 strokes. The homogenates were centrifuged at 750 *g *for 10 min at 4°C to remove the nuclei. Supernatants were centrifuged at 10,000 *g *for 15 min at 4°C. The crude mitochondrial pellet fractions were dissolved in Western blot sample buffer, and the supernatants were mixed with 2× sample buffer. For caspase cleavage analysis, enriched mitochondria were resuspended in 20 μl of buffer A and incubated for 1 h with 1 unit of recombinant human caspase 3 or caspase 8 (Millipore, Schwalbach, Germany).

## Results

### Nelfinavir induces apoptosis in human leukemia cells at concentrations that have limited effects on normal bone marrow cells

**Figure 1:**
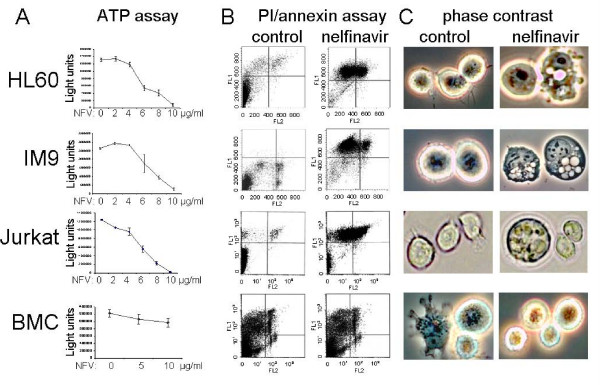
**Nelfinavir induces cell death in leukemia cells**. The human leukemia cell lines HL60, Jurkat, and IM9, and an *ex vivo *sample of human bone marrow cells (BMC) were treated for 48 h in cell culture with the indicated concentrations of nelfinavir and analyzed by a chemiluminescent ATP assay for cell survival (A), by annexin/PI staining [FL-1 (propidium iodide): 575 nm; FL-2 (FITC): 530 nm] for the occurrence of apoptosis and necrosis (B), and by phase contrast microscopy for morphological changes (C). For FACScan analysis and phase contrast microscopy, all cells were treated equally for 24 h with 8 μg/ml nelfinavir.

The human leukemia cell lines HL60 (promyelocytic leukemia), IM9 (EBV-transformed B-lymphoblastoid cell line) and Jurkat (T cell leukemia) were incubated with nelfinavir at concentrations between 0 and 10 μg/ml. Cell survival was then analyzed by a chemiluminescent ATP assay (Fig. 1). At concentrations between 4 and 10 μg/ml, nelfinavir induced cell death in all three leukemia cells tested, showing an ED50 (effective dose that reduces ATP activity by 50%) of 5.6-7 μg/ml and an ED90 of 9-10 μg/ml, depending on the cell line tested (Table 1). In human bone marrow cells (BMC) tested *ex vivo *under the same conditions, 10 μg/ml nelfinavir had only a slight effect on cell survival. However, BMC were not completely unaffected by nelfinavir, and higher nelfinavir concentrations (Table 1) were indeed able to induce BMC cell death. In leukemia cells treated with 8 μg/ml nelfinavir, phase contrast microscopy revealed extensive intracellular vacuole formation (Fig. 1), which was absent in BMC treated with the same nelfinavir concentration. To analyze the nature of nelfinavir-mediated cell death, a propidium iodide permeability (PI) and annexin binding assay was performed. FACScan analysis showed that a concentration of 8 μg/ml nelfinavir induced a significant increase in the number of apoptotic (annexin-positive) and necrotic or dead (PI-permeable) leukemia cells (Fig. 1), but had no detectable effects on the morphology or apoptosis of the rather heterogeneous BMC cell population (Fig. 1).

**Table 1:** Effective drug doses for nelfinavir-mediated cell death [μg/ml]

	HL60	Jurkat	IM9	BMC
ED50	5.6	5.8	7	15.2
ED80	8.9	7.8	9.3	23
ED90	9.8	9	10	N.D.

### Nelfinavir downregulates cyclin B and cdk1 expression and interferes with cell cycle progression

**Figure 2:**
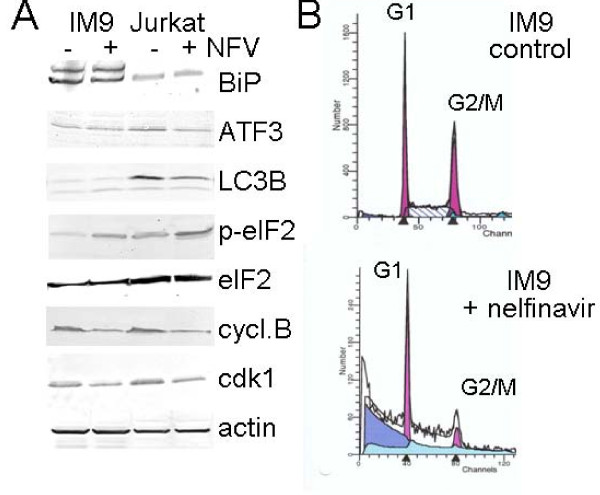
**Influence of nelfinavir on cell cycle progression and apoptosis in human leukemia cells**. IM9 and Jurkat cells were treated for 24 h with 8 μg/ml nelfinavir and analyzed by (A) Western blot analysis for the expression of cell stress- and cell cycle-related proteins and by (B) FACScan analysis for cell cycle distribution of nelfinavir-treated IM9 cells.

It has previously been shown by both our group [8] and others [1,2] that nelfinavir induces the endoplasmic reticulum stress response in solid human cancer cells, resulting in upregulation of BiP, phosphorylation of eIF2, upregulation of ATF3, and autophagy. In contrast to our results for ovarian cancer cells [8], Western blot analysis did not shown upregulation of BiP or ATF3 in nelfinavir-treated leukemia cells, and cells exhibited no signs of autophagy as shown by a lack of LC3B upregulation (Fig. 2A). However, nelfinavir induced a slight increase in eIF2 phosphorylation (Fig. 2A), suggesting an influence on cell cycle progression, which was further indicated by reduced expression of cyclin B and cdk1 (Fig. 2A). Cell cycle analysis by FACScan revealed a reduced G2/M peak, suggesting interference with cell cycle progression (Fig. 2B). However, the most prominent effect of nelfinavir appeared to be the induction of apoptosis, as indicated by a significant increase in the number of cells in the sub-G1 phase (Fig. 2B).

### Nelfinavir induces caspase activation and mcl-1 upregulation despite partial caspase 8-mediated mcl-1 cleavage

**Figure 3:**
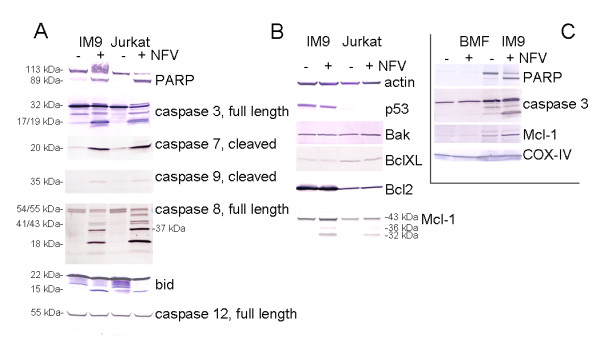
**Nelfinavir induces activation of caspase 3 and caspase 8 despite mcl-1 upregulation**. IM9, Jurkat and bone marrow fibroblast cells (BMF) were treated for 24 h with 8 μg/ml nelfinavir and analyzed by Western blot analysis for the expression of caspases and apoptosis-relevant proteins. (A) and (B): cell lysates of IM9 and Jurkat cells; (C) cell lysates of IM9 and BMF.

To gain better insight into the mechanism by which nelfinavir induced apoptosis and the extent of caspase involvement, we performed Western blot analysis for several apoptosis-related proteins. In accordance with the FACS analyses presented in Figs. 1 and 2B, induction of apoptosis by nelfinavir was confirmed by cleavage of PARP, a specific substrate of effector caspases 3 and 7, whose activation is shown by the appearance of their specific cleavage products (Fig. 3A). Caspases 3 and 7 are cleaved and activated by initiator caspase 9. Caspase 9 cleavage was observed in nelfinavir-treated leukemia cells by Western blot analysis, but the bands were rather faint (Fig. 3A). In contrast, significant activation of initiator caspase 8 was observed, suggesting potential involvement of an additional, mitochondria-independent apoptotic pathway. Activation of caspase 12, an initiator caspase downstream of ER stress, was not detected by Western blot analysis (Fig. 3A).

**Figure 4:**
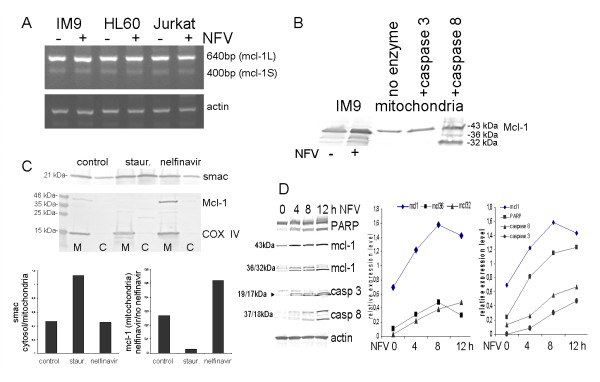
**Nelfinavir induces mitochondria protection despite caspase 8-mediated mcl-1 cleavage**. A) The indicated leukemia cell lines were treated with 8 μg/ml nelfinavir for 24 h hours and subjected to PCR analysis to detect potential mcl-1 splice forms and transcriptional mcl-1 regulation. B) An enriched cellular mitochondria fraction of IM9 cells was treated with recombinant caspase 3 or caspase 8 as indicated and subjected to Western blot analysis for mcl-1 expression. Whole cell extracts of nelfinavir-treated IM9 cells were run in parallel for comparison. C) IM9 cells treated for 24 h with 8 μg/ml nelfinavir or 500 nM staurosporine were separated into a crude mitochondria fraction (M) and a crude cytosol fraction (C) and analyzed by Western blot for the distribution of mcl-1, smac and mitochondria-resident protein cytochrome c oxidase (COX IV). For direct comparison, mcl-1 and COX IV immunostaining were performed on the same blot. Staining intensities were analyzed with a BioRad gel documentation system using the BioRad Quantity One program (BioRad, Munich, Germany) and expressed as relative expression values. D) IM9 cells were treated with 10 μg/ml nelfinavir for the indicated times and analyzed by Western blot analysis. Selected bands (mcl1: 43, 36, 32 kDa; caspase 3: 17+19 kDa; caspase 8: 18 kDa) were densitometrically analyzed using the BioRad Quantity One program and plotted as relative expression values versus β-actin expression.

To further investigate the mechanism leading to nelfinavir-induced apoptosis, the expression of several apoptosis-regulatory proteins was analyzed. Nelfinavir did not increase the expression of p53 in IM9 cells (Fig. 3A). In addition, the expression of the small bcl family members, bak, bcl-XL and bcl2, appeared to be unchanged (Fig. 3A). Unexpectedly, however, we observed an upregulation of the anti-apoptotic mcl-1 protein in nelfinavir-treated cancer cells (Fig. 3A). Upregulation of mcl-1 by nelfinavir occurred in leukemia cells, but not in bone marrow fibroblasts (BMF) generated from bone mesenchymal marrow cells by cell culture propagation (Fig. 3B). In addition to the accumulation of full-length mcl-1 (43 kDa), shorter mcl-1 immunoreactive bands appeared in nelfinavir-treated leukemia cells (Fig. 3A, B), representing either splice variants or cleavage products of mcl-1. To distinguish the relative expression levels of the mcl-1 splice variants, we performed RT-PCR analysis, which revealed that anti-apoptotic mcl-1L is the most prominent form expressed by leukemia cells. In contrast, the pro-apoptotic mcl-1S form, generated by internal alternative splicing, was poorly expressed and was not upregulated by nelfinavir treatment (Fig. 4A). In order to demonstrate that the shorter forms of mcl-1 could represent mcl-1 cleavage products and not the splice variant mcl-1S, mitochondria enriched by cellular subfractionation of IM9 cells were prepared and incubated with recombinant caspase 3 and caspase 8. The addition of purified caspase 8 but not caspase 3 to the mitochondria resulted in the formation of mcl-1 cleavage products that were identical to those obtained by incubation of viable IM9 cells with nelfinavir (Fig. 4B). Thus, the additional bands presenting mcl-1 immunoreactivity observed after nelfinavir treatment represent mcl-1L degradation products and not the pro-apoptotic short splice form of mcl-1, mcl-1S.

### Nelfinavir induces mitochondria protection in leukemia cells

**Figure 5:**
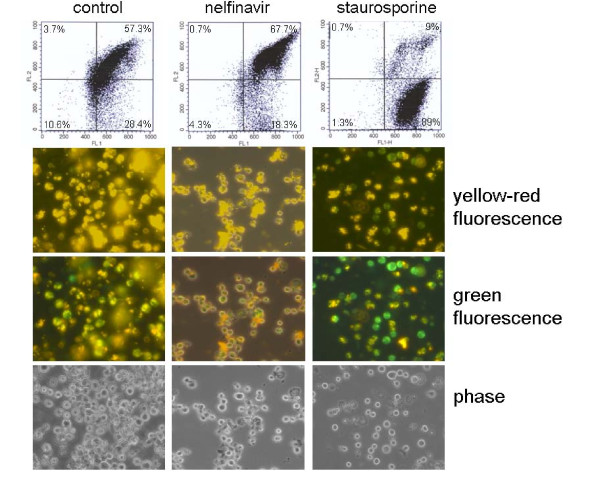
**Nelfinavir-induced cell death is independent of mitochondrial membrane depolarization**. IM9 cells were treated for 24 h with 8 μg/ml nelfinavir or 500 nM staurosporine and the outer mitochondrial membrane potential was analyzed using the MitoCapture kit (Alexis, Lörrach, Germany) by either FACScan analysis or fluorescence microscopy.

In standard apoptotic conditions, pro-apoptotic bcl-2 family members such as bak or t-bid insert into the outer mitochondrial membrane and induce pore formation, resulting in the efflux of mitochondrial proteins such as cytochrome c and smac/DIABLO. The efflux of smac into the cytosol can be monitored experimentally by cell fractionation studies. In IM9 cells, the classical apoptosis-inducer staurosporine caused an accumulation of smac in the cytosol, accompanied by downregulation of mcl-1 (Fig. 4C). In contrast, nelfinavir treatment of IM9 cells enhanced mitochondrial mcl-1 expression and had no effect on the cellular distribution of smac (Fig. 4C). These results were confirmed using a fluorescent mitochondria tracker dye that accumulates within intact mitochondria as a red-fluorescent dye or within the cytosol as a monomer that exhibits green fluorescence. Both FACScan and fluorescence analysis showed that the mitochondrial membrane potential of IM9 cells is disrupted by staurosporine but not by nelfinavir treatment (Fig. 5). Even more, the percentage of cells with intact mitochondrial membrane potential appeared to be increased after nelfinavir treatment (Fig. 5). A time-dependent analysis of the expression of pro- and anti-apoptotic proteins in nelfinavir-treated IM9 cells revealed a rather immediate upregulation of mcl-1 after nelfinavir treatment, and a continuous and obviously concomitant increase in caspase and PARP cleavage products (Fig. 4D). At later stages of apoptosis, the 36 kDa mcl-1 cleavage product appeared to be further converted into a 32 kDa cleavage product (Fig. 4D).

### Sorafenib downregulates mcl-1 expression and enhances nelfinavir-mediated cell death of leukemia cells

**Figure 6:**
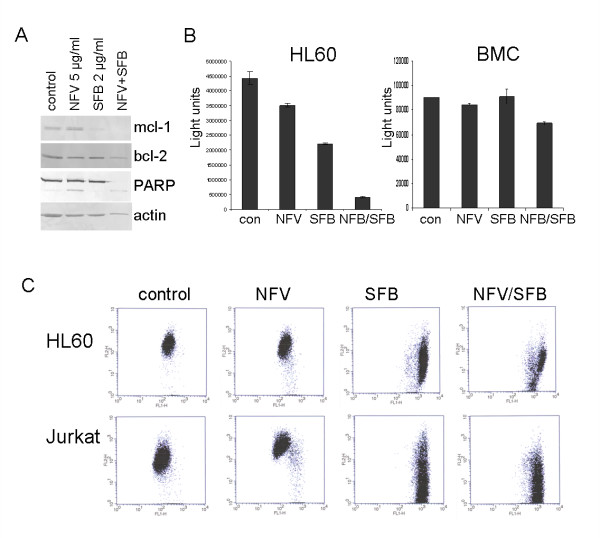
**Sorafenib enhances the efficacy of nelfinavir-induced cell death by mcl-1 downregulation**. A) HL60 cells were treated with 5 μg/ml nelfinavir or 2 μg/ml sorafenib either alone or in combination, and analyzed after 24 h for the expression of apoptosis-related proteins by Western blot analysis. B) HL60 cells and *ex vivo *bone marrow cells (BMC) were incubated using the same conditions as in (A) and analyzed after 48 h for total cell survival by an ATP assay. C) HL60 and Jurkat cells were treated with either 5 μg/ml nelfinavir or 2 μg/ml sorafenib either alone or in combination, and the outer mitochondrial membrane potential was analyzed after 24 h by FACScan analysis.

Because the previous experiments revealed that nelfinavir induced a mitochondria-independent apoptotic pathway, we tested whether pharmacological downregulation of mcl-1 could further enhance the cytotoxic effect of nelfinavir on leukemia cells by additionally activating the mitochondrial pathway. The multikinase inhibitor sorafenib, an approved drug for the treatment of renal cancer, has been shown to downregulate the expression of mcl-1 at both the transcriptional and posttranscriptional level [21]. Fig. 6A shows that at a concentration of 2 μg/ml, sorafenib efficiently reduced mcl-1 expression in HL60 cells, with little effect on bcl-2 expression. When combined with 5 μg/ml nelfinavir, a concentration that inefficiently induces cell death when applied alone (Fig. 6B), sorafenib significantly enhanced the efficacy of nelfinavir. In addition, FACScan analysis showed that sorafenib alone or in combination with nelfinavir leads to a loss of outer mitochondrial membrane potential (Fig. 6C). To exclude the possibility that this drug combination is potentially myelosuppressive, we tested nelfinavir in combination with sorafenib on bone marrow cells *ex vivo*. The same dose of nelfinavir and sorafenib that caused significant cell death in leukemia cells had only limited effects on bone marrow cells (Fig. 6B).

## Discussion

Mcl-1 is a crucial regulator of cell death in leukemia cells [18]. Overexpression of mcl-1 can inhibit cell death by stabilizing the outer mitochondrial membrane potential [9-14], and several recent leukemia treatment strategies have attempted to target the expression of mcl-1 by either pharmacological inhibition or siRNA-mediated downregulation [22]. Our investigations show that nelfinavir, despite its ability to induce death of leukemia cells, induces an upregulation of the cell-protective mcl-1 protein in human leukemia cells that might stabilize the mitochondria even under apoptotic conditions. Because we did not observe increased mcl-1 mRNA expression by RT-PCR analysis, and the mcl-1 protein was upregulated within hours, mcl-1 is probably stabilized by posttranscriptional mechanisms. We have recently shown that the mcl-1 protein can be stabilized in solid cancer cells by ERK1/2-mediated protein phosphorylation [23]. However, we could not detect activation of this pathway in leukemia cells (data not shown), suggesting that other mcl-1 protein stabilization mechanisms may function in leukemia cells.

Nelfinavir has previously been observed to have both cell- and tissue-protective effects on various human and murine cells and tissues [24-27]. For example, in contrast to the pro-apoptotic effect of nelfinavir on leukemia cells, it is cytoprotective for murine liver cells [25], neurons [25], retina cells [26], and pancreas cells [27]. Interestingly, the cytoprotective effect of nelfinavir has already been associated with mitochondria protection [24,25]. Upregulation of mcl-1 could be involved in nelfinavir-mediated cytoprotection of several untransformed cell types, although we did not observe significant endogenous mcl-1 expression or even nelfinavir-induced mcl-1 upregulation in bone marrow fibroblasts (Fig. 2) or leukocytes (data not shown). In some previous studies, the mitochondria-protective effect of nelfinavir was found to be independent of protein synthesis [24] and to be mediated by direct binding of nelfinavir to the adenine nucleotide translocase (ANT) [24,25], a subunit of the mitochondrial permeability transition pore complex [28,29]. Thus, nelfinavir-mediated mitochondria-protection and cell death can be modulated by various mechanisms that might vary among cell types and species. Interestingly, a similar paradoxical effect has been observed for glucocorticoids (dexamethasone), which induce apoptosis in leukemia cells but protect normal and cancerous epithelial cells by upregulating anti-apoptotic proteins [30,31]. However, the prospect of nelfinavir as a multipotent cytoprotective agent with selective anti-cancer activity should be considered with caution and may be an unachievable benchmark for this drug. We have observed that higher doses of nelfinavir can indeed induce cell damage in human bone marrow cells (Table 1) and, thus, nelfinavir should not be regarded as a bone marrow-protective drug. Still, the nelfinavir concentration necessary to induce high levels of apoptosis in leukemia cells showed only a limited effect on bone marrow cells, thus providing a potential therapeutic concentration for efficient leukemia treatment with reduced adverse effects on the bone marrow. This is especially important given that the bone marrow is already damaged in leukemia patients after standard first- and second-line high dose chemotherapies with myelosuppressive drugs.

These data, as well other reports, indicate that the concentration of nelfinavir appears to be of crucial importance for its effect as either a cytoprotective drug or a cell death-inducing agent. In HIV-infected persons treated with nelfinavir, individual nelfinavir plasma concentrations were found to be highly variable, with a mean average drug plasma concentration of 2.22 ± 1.25 μg/m [32]. This level is below the concentration that induces death of leukemia cells or other cancer cells. In fact, a recent study on the occurrence of cancer in nelfinavir-treated HIV patients revealed no reduced cancer risk [33], confirming that these concentrations are sub-optimal for cancer treatment. However, the plasma concentrations occurring in HIV patients have been specifically adapted for efficient and long term HIV protease inhibition. Administering higher oral doses of nelfinavir or applying nelfinavir via an intravenous route can significantly enhance plasma nelfinavir concentrations [34]. Further, and more likely in the potential clinical use of nelfinavir for cancer therapy, efficient combination treatments with other drugs may allow the effective concentration of nelfinavir to be reduced, as shown in the present *in vitro *study through the combination of nelfinavir and sorafenib.

## Conclusions

The results obtained by our group and others show that nelfinavir could become a potential and valuable new anti-cancer drug, not only because of its anti-cancer effects *in vitro *and *in vivo*, but also because of its proven pharmacological history and known and tolerable side effects [35,36]. Therefore, we strongly recommend clinical studies with nelfinavir in leukemia patients, preferentially in combination with sorafenib.

## Competing interests

The authors declare that they have no competing interests.

## Authors' contributions

AB and MR performed the experiments, and AG and KF provided necessary reagents. All authors read and approved the final manuscript.
